# Recent Advances in the Molecular Beacon Technology for Live-Cell Single-Molecule Imaging

**DOI:** 10.1016/j.isci.2020.101801

**Published:** 2020-11-13

**Authors:** Shiqi Mao, Yachen Ying, Ruonan Wu, Antony K. Chen

**Affiliations:** 1 Department of Biomedical Engineering, College of Engineering, Peking University, No. 5 Yiheyuan Road, Haidian District, Beijing 100871, China

**Keywords:** Molecular Genetics, Biodevices, Nanotechnology

## Abstract

Nucleic acids, aside from being best known as the carrier of genetic information, are versatile biomaterials for constructing nanoscopic devices for biointerfacing, owing to their unique properties such as specific base pairing and predictable structure. For live-cell analysis of native RNA transcripts, the most widely used nucleic acid-based nanodevice has been the molecular beacon (MB), a class of stem-loop-forming probes that is activated to fluoresce upon hybridization with target RNA. Here, we overview efforts that have been made in developing MB-based bioassays for sensitive intracellular analysis, particularly at the single-molecule level. We also describe challenges that are currently limiting the widespread use of MBs and provide possible solutions. With continued refinement of MBs in terms of labeling specificity and detection accuracy, accompanied by new development in imaging platforms with unprecedented sensitivity, the application of MBs is envisioned to expand in various biological research fields.

## Graphical Abstract

**Figure:**
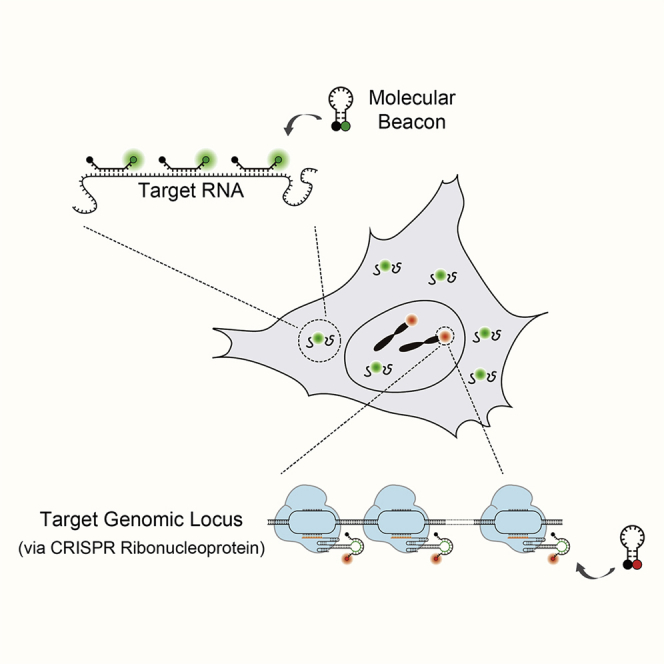

## Teaser

Subject Areas: Molecular Genetics; Biodevices; Nanotechnology

## Introduction

**Figure 1:**
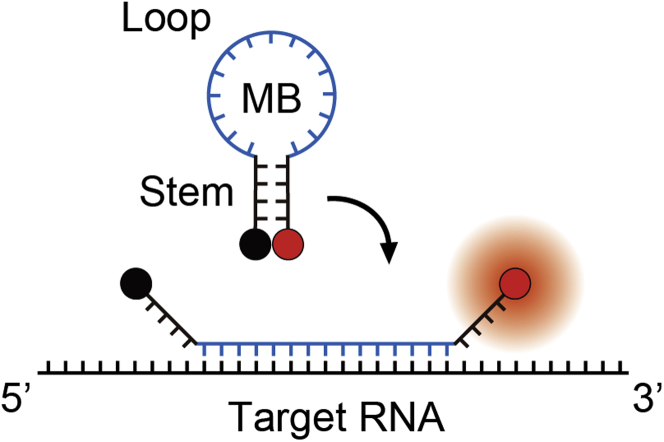
Schematic of an MB in the Absence or Presence of a Target RNA An MB generally comprises a loop containing 15–25 nucleotides and a stem containing 4–7 base pairs. In the absence of a target RNA, it remains in the stem-loop configuration whereby the fluorophore (red circle) and the quencher (black circle) are held in close proximity. Hybridization of a target RNA to the loop domain causes separation of the two labels to restore MB fluorescence.

Nucleic acids are biological polymers with unique properties such as specific base pairing, well-defined composition, and predictable structure and size, making them ideal materials for engineering artificial nanodevices that can interface with the biological world (Chakraborty et al., 2016; Jasinski et al., 2017; Samanta et al., 2020). For live-cell labeling of native RNA transcripts, the majority of the nanodevices currently in use function via antisense-based recognition, i.e., using single-stranded oligonucleotides to hybridize with specific sequences within the target RNA of interest (Bao et al., 2009; Tyagi, 2009; Mannack et al., 2016; George et al., 2018; Braselmann et al., 2020), with the most widely employed nanodevice to date being the molecular beacon (MB), an invention of Tyagi and Kramer in 1996 that represents a class of stem-loop-forming hybridization-activated oligonucleotide probes bearing an organic dye (fluorophore) at one end and a quencher at the other (Tyagi and Kramer, 1996) (Figure 1). In the inactive state, MBs are significantly quenched because the fluorophore and the quencher are held in juxtaposition by the complementary short arm sequences at the ends that anneal to form a stable duplex stem. Hybridization of the unique MB target sequence to the loop domain overcomes the energy barrier imposed by the stem, leading to stem unwinding and ultimately separation of the two labels to restore MB fluorescence. The useful fluorogenic properties, the small size (~10 kDa), and the simple yet effective means to report target recognition have led to extensive applications of MBs for live-cell detection of specific RNAs in various biological and pathological contexts (Bratu et al., 2003; Tyagi and Alsmadi, 2004; Medley et al., 2005; Peng et al., 2005; Vargas et al., 2005; Santangelo and Bao, 2007; Wang et al., 2008; Yeh et al., 2008; Kang et al., 2011; Yang et al., 2011; Jha et al., 2015; Turner-Bridger et al., 2018; Cioni et al., 2019; Oliveira et al., 2020). Moreover, MBs are highly amenable to modification and have thus far served as prototypes for more advanced MB architectures such as MBs that incorporate bright and photostable nanocrystals in place of the standard organic dye labels (Kim et al., 2004) or wavelength-shifting MBs that incorporate an additional dye (i.e., a harvester fluorophore) to enable multiplexed genetic analyses via a monochromatic light source (Tyagi et al., 2000).

Although MBs have shown great promise for intracellular RNA analysis, their potential has not yet been fully realized, as the majority of existing MB-based assays rely on bulk fluorescence measurements, which are suitable for visualizing highly abundant RNA but cannot reveal the spatiotemporal heterogeneity among individual RNA transcripts, information that is crucial for better characterization of the molecular basis of human health and disease (Raj and van Oudenaarden, 2009; La Manno et al., 2018; Xia et al., 2019). Given that the most widely used method for studying single-molecule RNA dynamics, i.e., the MS2 system (Ben-Ari et al., 2010; Larson et al., 2011; Wu et al., 2012), demands genetic insertion to tag the RNA under investigation and is based on fluorescent protein (FP) reporters that generally lack sufficient brightness and photostability to allow continuous imaging, there has been a great interest in developing single-molecule imaging methods using MBs, as the probes are capable of labeling unmodified endogenous transcripts and comprise fluorophores that are generally brighter and more photostable than FPs.

Until now, several reviews have described MB design fundamentals guided by hybridization kinetics and thermodynamics-based analysis, together with their live-cell applications based on bulk fluorescence (Goel et al., 2005; Bratu, 2006; Bao et al., 2009; Chen and Tsourkas, 2009; Bratu et al., 2011; Monroy-Contreras and Vaca, 2011; Yang and Tan, 2013; Zheng et al., 2015). Additionally, an excellent and comprehensive database containing various aspects of MBs, including probe design fundamentals, synthesis methods, and the applications both in solution and in cells, is available at http://www.molecular-beacons.org/MB_publications.html#cap1. More recently, reviews that overview the field of single-molecule imaging methods have briefly outlined the capacity of MBs for illuminating RNA activities at the single-molecule level (Elf and Barkefors, 2019; Kim et al., 2019; Sato et al., 2020). In this review, emphasis is given to the progress made in advancing the MB technology toward live-cell single-molecule analysis. We begin by summarizing methods for intracellular delivery of MBs. This is followed by an outline of existing strategies to minimize the incidence of MB false-positives arising from nonspecific protein binding and nuclease degradation. Furthermore, we describe the progress made in MB-based single-molecule imaging. Finally, several issues that may hamper widespread use of MBs and their potential solutions are discussed.

## Cellular Delivery of MBs

Similar to many nucleic acid-based nanodevices, MBs cannot move freely across the negatively charged plasma membrane without assistance. Nanoparticles or cell-penetrating peptides that can enter the cells via endocytosis have served as popular vehicles for MB delivery. However, it has been observed that a considerable amount of MBs following the uptake can ultimately localize and accumulate within late endosomes and lysosomes (Chen et al., 2008), making RNA labeling in other regions difficult. Although polymers such as polyethylenimine (Akinc et al., 2005) capable of causing osmotic swelling-mediated rupture of endosomes and lysosomes have been used to promote the escape of MBs (Dong et al., 2011; Wang et al., 2016), whether the release is profound to permit effective RNA labeling still remains elusive. Therefore, methods that can directly introduce MBs into cells without the involvement of endocytosis are generally preferred.

**Figure 2:**
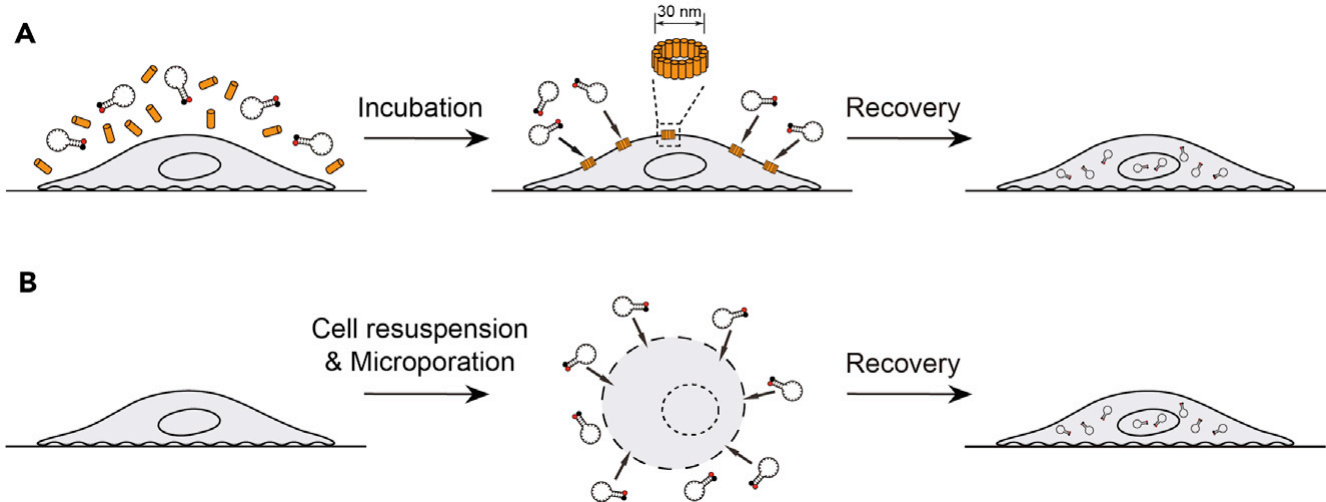
Schematic of Delivering MBs into Living Cells Using SLO or Microporation (A) SLO. Adherent cells are first incubated with a mixture containing SLO (orange columns) and MBs. Following formation of SLO pores (with diameters of ~30 nm) on the plasma membrane and entry of MBs into the cells through the pores, the plasma membrane can be sealed by replacing the SLO/MB-containing media with normal cell culture media. (B) Microporation. Adherent cells are first resuspended in electroporation buffer containing MBs. During microporation, MBs enter the cells through the pores generated by brief electrical pulses. After microporation, cells are incubated in normal cell culture media and seeded onto a surface pre-coated with extracellular matrix proteins, which may facilitate cell adhesion.

Currently, the most straightforward method for delivering MBs into cells is microinjection, which uses a glass micropipette with a fine tip having an inner diameter of 0.5–1 μm to inject a small volume of aqueous solution containing the sample of interest into cells. Microinjection offers the benefits of precise delivery and instantaneous monitoring, but is labor-intensive and technically demanding, making high-throughput RNA analysis impractical. To deliver MBs into cells more efficiently, several research groups make use of the highly repairable nature of the plasma membrane for small lesions. One approach uses streptolysin-O (SLO), a bacterial exotoxin that interacts with cholesterol, to form pores of 30 nm in size at the plasma membrane (Palmer et al., 1998). It has been reported that molecules with molecular weight less than 150 kDa can traverse the SLO pores without apparent restriction (Walev et al., 2001; Teng et al., 2016). In a typical experiment, the cells are first incubated with a mixture containing MBs and SLO previously activated using reducing agents (Figure 2A). After sufficient time is given to allow MBs to enter the cells, the mixture is replaced with normal cell culture media to seal the plasma membrane. Depending on the cell type, up to 100% delivery efficiency may be achieved (Santangelo et al., 2006). However, due to the toxicity of SLO, high probe delivery efficiency may be accompanied by low cell viability, making rigorous optimization of experimental parameters such as SLO/probe concentration, SLO/probe incubation time, and cell seeding density necessary. A second approach utilizes electroporation, which relies on applying short high-voltage pulses to generate short live pores to enable instantaneous delivery (Kim et al., 2008; Lee et al., 2009; Giraldo-Vela et al., 2015). One promising system is microporation, which utilizes a μL-volume pipette tip as an electroporation chamber to generate a highly uniform electrical field that largely avoids the problems of conventional electroporation systems including pH variation, oxide formation, temperature elevation, and metal ion generation (Kim et al., 2008; Lee et al., 2009). In a typical procedure, cells are first resuspended in a small volume of an electroporation buffer containing MBs (Figure 2B). Following microporation, the cells are subjected to a recovery step where they are incubated in normal cell culture media and seeded on surfaces pre-coated with extracellular matrix proteins that facilitate cell adhesion. Although a special equipment is required and the consumables are relatively costly, microporation could achieve nearly 100% MB delivery with >85% viability for various cell types (Chen et al., 2008; Zhao et al., 2016).

## Minimizing Nonspecific MB Signals *In Vivo*

**Figure 3:**
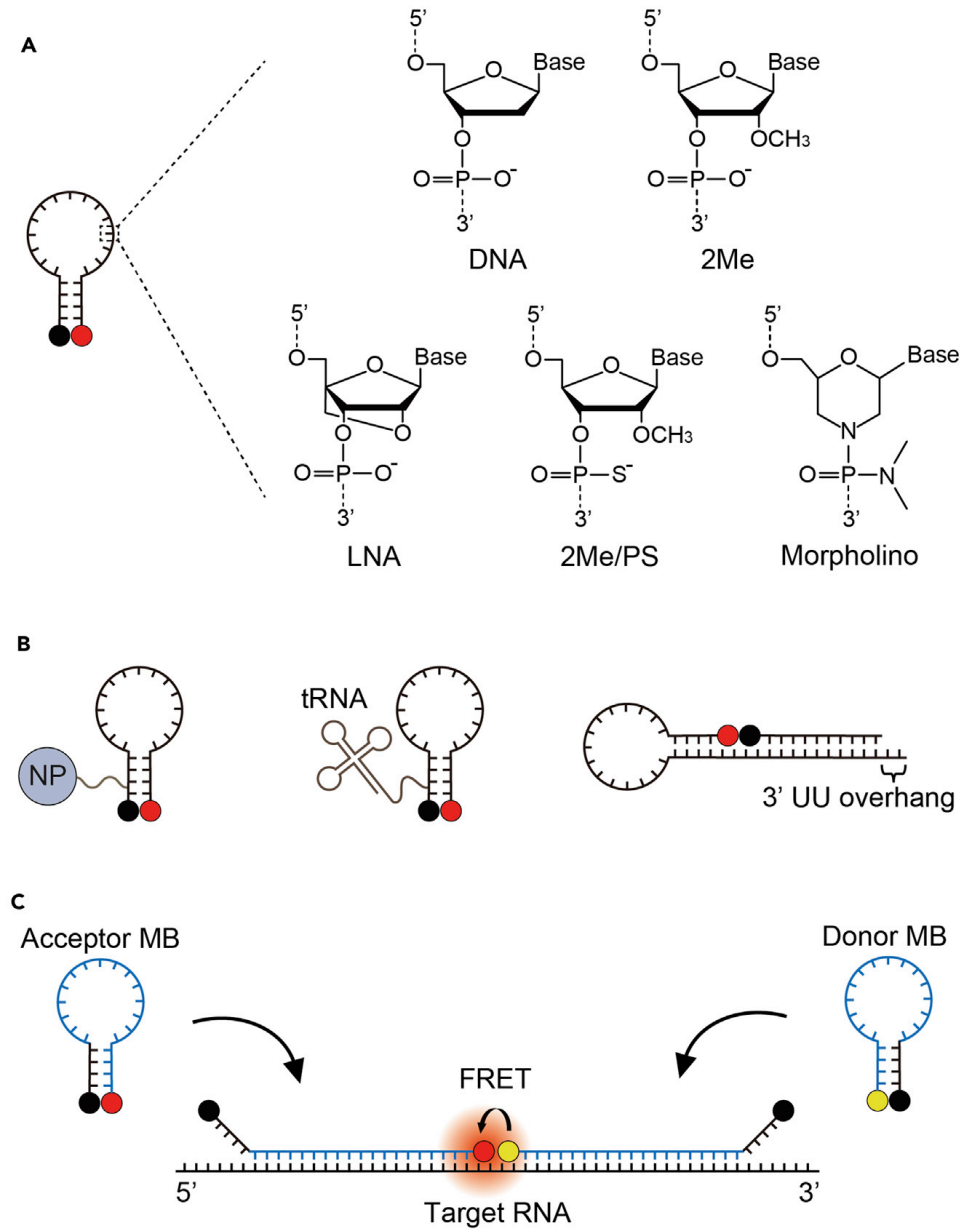
Strategies for Minimizing MB False-Positive Signals in Living Cells (A) Improving MB biostability. An MB may be synthesized with a backbone partially or entirely modified with unnatural nucleotide analogs (i.e., LNA nucleotides, 2Me/PS nucleotides, or morpholinos) to confer the MB improved biostability compared with an MB whose backbone is composed of DNA or 2Me nucleotides only. (B) Minimizing MB nuclear entry. An MB can be attached to a nanoparticle (NP), a tRNA molecule, or an siRNA-like element (i.e., a duplex of 18 base pairs with a 3′ UU-overhang). (C) Dual-FRET MB. Collective hybridization of the donor and acceptor MBs to adjacent regions on the same target RNA leads to generation of a FRET signal as the donor fluorophore (yellow circle) and the acceptor fluorophore (red circle) are held in close proximity. Shared-stem MBs (i.e., both the loop and one arm of the stem participate in target hybridization) are typically used because the relative distance between the donor and acceptor fluorophores can be fixed to yield a stable FRET signal. The MB sequences that participate in target hybridization are shown in blue.

Although the majority of existing MB-based imaging studies have employed MBs synthesized with naturally occurring DNA (DNA MBs) or 2′-*O*-methyl RNA (2Me MBs) nucleotides, it is now fairly clear that both types of MBs are highly prone to nonspecific opening caused by nuclease digestion or nonspecific protein binding inside cells, leading to the generation of false-positives. To circumvent this problem, one widely used approach has been to synthesize the MB backbone with unnatural nucleotide analogs that have been developed for protecting antisense agents from nuclease digestion or nonspecific protein binding (Chan et al., 2006) (Figure 3A). Locked nucleic acid (LNA) nucleotide is one such analog, in which the ribose moiety is locked into a rigid C3′-endo conformation by a simple 2′-O, 4′-C methylene bridge (Petersen and Wengel, 2003). As LNA nucleotides have high binding affinity for each other at the physiological temperature (Koshkin et al., 1998; Vester and Wengel, 2004), a fully LNA-modified MB may not hybridize to the target RNA effectively (Wang et al., 2005; Yang et al., 2007). As such, for live-cell imaging, chimeric MBs composed of LNA with DNA or 2Me nucleotides have always been used. In the former case, MBs have been mostly designed to contain a stem comprising 50% LNA in an alternating fashion and a loop comprising 50%–100% LNA (Wu et al., 2008). In the latter case, MBs have been designed with a stem completely free of LNA and a loop containing LNA nucleotides separated by at least one 2Me nucleotide (Catrina et al., 2012; Turner-Bridger et al., 2018; Cioni et al., 2019), a modification strategy unique to LNA/2Me chimeras that can enhance the stability of the duplex formed with RNA (Kierzek et al., 2005). Alternative to modifying the sugar moiety, a non-bridging oxygen of the phosphate group has been substituted with a sulfur atom to form the nuclease-resistant phosphorothioate (PS) internucleotide linkage (Matsukura et al., 1987; Yeh et al., 2008; Chen et al., 2009; Zhao et al., 2016). Although incorporating PS throughout the backbone can render MBs highly nuclease resistant, the extensive PS modification can also cause MBs to be highly prone to forming aggregates as previously observed with extensively PS-modified oligonucleotides in the nucleus (Lorenz et al., 1998), emitting non-negligible false-positive signals that may be misinterpreted as single or clusters of RNA transcripts (Chen et al., 2009; Zhao et al., 2016). We have recently optimized the extent of PS modification on MBs and showed that a design in which only the loop domain is PS-modified (i.e., 2Me/PS_LOOP_ MB) can exhibit reduced nonspecific binding while maintaining nuclease resistance in various cell types (Zhao et al., 2016). Finally, MBs have also been synthesized with morpholino, in which the deoxyribose/ribose moiety and the phosphate group are substituted with a morpholine ring and a non-ionic phosphorodiamidate internucleotide linkage, respectively (Summerton and Weller, 1997; Chen et al., 2016). It should be noted that, for any type of chemically modified MB, synthesis could be complicated to result in a low product yield compared with DNA or 2Me MBs.

Besides backbone modification, based on studies showing that MBs are highly prone to nuclear sequestration and the nucleus is the primary region where MB nonspecific opening occurs (Tyagi and Alsmadi, 2004; Mhlanga et al., 2005; Chen et al., 2007), several groups have demonstrated successful reduction of false-positive signals of MBs by minimizing their entry into the nucleus (Figure 3B). For example, MBs may be linked to nanoparticles such as quantum dots (Chen et al., 2007), which are too large (~27 nm in diameter) to traverse nuclear pore channels that are only ~5 nm in diameter (Cady et al., 2007; Jena, 2020). Another approach involves tethering MBs to mimics of small RNAs such as transfer RNA (tRNA) (Mhlanga et al., 2005) and small interfering RNA (siRNA) (Chen et al., 2010), which upon entering the nucleus are quickly exported by the nuclear export machineries into the cytoplasm. Compared with nanoparticles, the small RNA mimics could interfere less with MB delivery and the activities of MB-labeled RNAs. Nonetheless, before cytoplasmic retention, a considerable amount of MB-small RNA conjugates could transiently localize in the nucleus (e.g., for 30 min) and thus could be engaged in nonspecific interaction temporarily.

Finally, false-positive signals may be minimized via the dual-fluorescence resonance energy transfer MB (dual-FRET MB) approach (Bratu et al., 2003; Tsourkas et al., 2003b; Santangelo et al., 2004), in which two distinct MBs, one labeled with a donor fluorophore at the 3′ end and the other labeled with an acceptor fluorophore at the 5′ end, are used to target adjacent regions on the same target RNA (Figure 3C). As FRET signal can only be generated when the two MBs are bound to the same target, this strategy can avoid essentially any nonspecific signals emitted by single MBs. However, successful imaging hinges upon the availability of a unique target region that is devoid of secondary structure and long enough to accommodate hybridization of both MBs, as well as careful donor/acceptor fluorophore selection to minimize the background fluorescence signal stemming from direct excitation of the acceptor fluorophore.

## MB-Based Dynamic Imaging of Single RNA Transcripts

MB-based imaging of RNA dynamics in living cells was first realized in 2003 by Bratu et al., who employed standard and dual-FRET 2Me MBs (denoted as binary MBs in the study) to label native *oskar* mRNAs in *Drosophila melanogaster* oocytes (Bratu et al., 2003). By following the movement of ribonucleoprotein (RNP) particles that could contain as many as 100 *oskar* mRNA molecules per particle from the nurse cell where they were produced to the posterior cortex of the oocyte where they were ultimately localized, the authors reported the first visualization of transient accumulation of the RNP particles in the center of the oocyte that lasted for ~25 min during the journey. These insightful results provided support for the idea that *oskar* mRNA may transiently accumulate in the center of an oocyte during oocyte maturation. However, because of the general difficulties in rendering individual fluorescent molecules perceptible in the cellular environment, important fundamental questions such as the mechanism driving the formation of single RNP particles from individual mRNA molecules could not be addressed. This prompted a number of research groups to develop MB-based imaging methods for visualizing RNA transcripts in living cells with single-molecule sensitivity.

**Figure 4:**
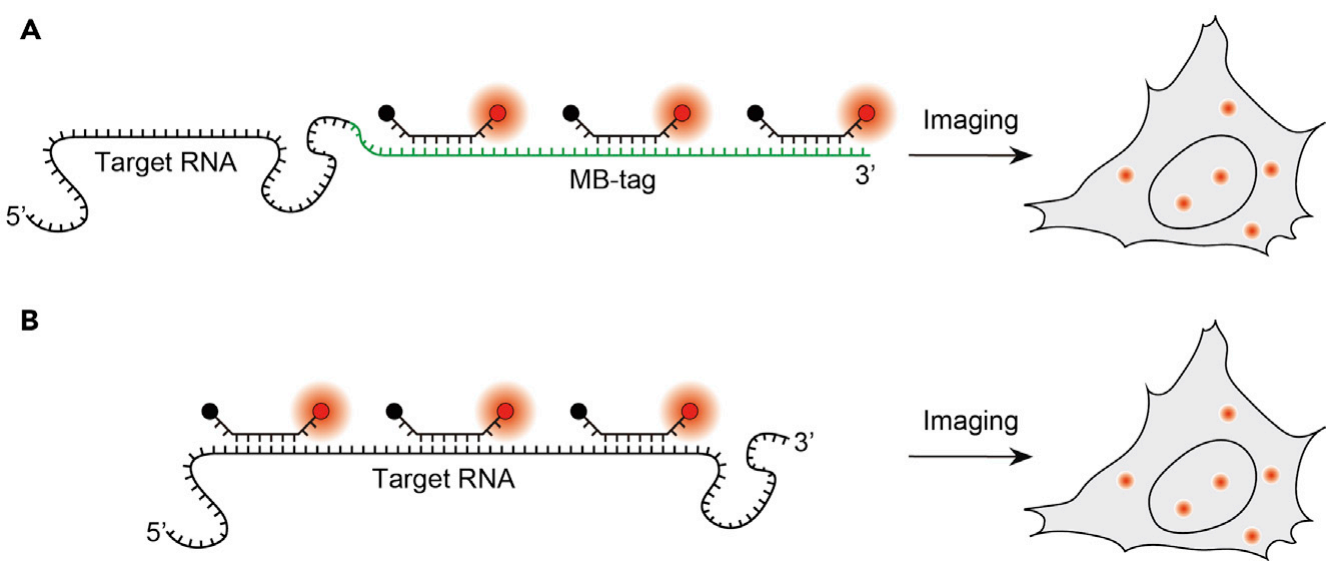
Illuminating Single RNA Transcripts Using MBs (A) In the MB-tag approach, a target RNA is engineered to harbor an MB-tag (green) containing multiple tandem repeats of a sequence that can be specifically bound by a unique MB. (B) In the direct labeling approach, multiple MBs are designed to bind to different regions of an unmodified endogenous target RNA. In both cases, collective hybridization of MBs to the target sites can illuminate the target RNA as a single bright spot.

To illuminate RNA transcripts at the single-molecule level, a general approach has been to employ multiple fluorescent probes to label a single RNA transcript, such that each labeled transcript appears as a bright spot under fluorescence microscopy. For MB-based single RNA imaging, one highly adoptable approach involves genetic modification of a target RNA with an MB-tag containing multiple copies of the same sequence that can hybridize to a single type of MB (i.e., the MB-tag approach) (Figure 4A). Alternatively, multiple distinct MBs may be used to label different regions of an unmodified endogenous RNA (i.e., the direct labeling approach) (Figure 4B). Below, the progress made in each approach and their applications in RNA studies are described.

The MB-tag approach was developed by Vargas et al., who engineered the green fluorescent protein (GFP) mRNA transcript to harbor 96 tandem repeats of a 50-nucleotide unique MB target sequence (Vargas et al., 2005). Using 2Me MBs to visualize the transcript expressed under the control of the doxycycline responsive TRE promoter, the authors studied the mechanism by which newly synthesized mRNA transcripts move through the dense nucleoplasm to reach the nuclear pores. It was found that the majority of mobile transcripts move freely within the nucleoplasm via Brownian diffusion, in contrast to the notion that the movement toward nuclear pores is energy-dependent and involves a chain of receptors (Agutter, 1994). Only when the molecules became stalled such as when wandering into the dense chromatin was ATP required to resume their motion. Additionally, their exit into the cytoplasm was shown to be independent of whether the transcription site is located near the nuclear periphery, in contrast to the theory that mRNA exits the nucleus through the nearest pores (Blobel, 1985). In a later work, Zhang et al. investigated the motion of the same engineered RNA in the cytoplasm using ratiometric bimolecular beacon, an MB that incorporates the functional element of an siRNA (Zhang et al., 2014). It was found that in contrast to the predominant Brownian motion in the nucleus observed by Vargas et al. (2005), the motion of the mRNA in the cytoplasm could switch among Brownian, sub-diffusive, and directed movements. More recently, we sought to quantify the impact of appending an MB-tag on the mobility of a target RNA by comparing the intracellular dynamics of engineered GFP mRNA transcripts harboring MB-tags of different sizes in cells using 2Me/PS_LOOP_ MB (Chen et al., 2017). Specifically, the mean diffusion rate of the transcript containing 32 repeats was nearly 10 times slower than that of the transcript containing 8 repeats (the limit of our microscopy setup) in both the nucleus and the cytoplasm, suggesting that intracellular activities of target RNAs are less impeded by smaller engineered insertions. We further showed that human Nuclear Enriched Abundant Transcript 1 (NEAT1) and HOX Transcript Antisense RNA (HOTAIR) long noncoding RNAs (lncRNAs) engineered to harbor the 8 repeats when labeled by 2Me/PS_LOOP_ MBs can exhibit single-molecule dynamics and localization that are physiologically relevant.

Compared with the MB-tag approach, the direct labeling approach avoids the need of appending any sequence, and thus should, in principle, impose less adverse impact on the activity of target RNA. However, the direct labeling approach could be more challenging, as RNA can be highly structured and bound by many RNA-binding proteins, making it difficult to identify a sufficient number of reliable, single-stranded regions accessible by MBs for single-molecule imaging via epifluorescence microscopy (at least 8 regions, as suggested by our finding based on the MB-tag approach, Chen et al. 2017). Consequently, imaging single unmodified endogenous RNAs has thus far been achieved using more advanced imaging techniques. Notably, Turner-Bridger et al. (2018) have reported successful imaging of single β-actin mRNA dynamics in growing axons with only two LNA/2Me MBs under highly inclined and laminated optical sheet microscopy, a single-molecule fluorescence technique that employs a highly inclined laser beam to illuminate a thin section of a specimen with a signal-to-background ratio approximately 8-fold greater than that of epi-illumination (Tokunaga et al., 2008). It was found that density distributions of the mRNAs are different in different axonal subcompartments. Moreover, the observed enrichment of the mRNAs at the growth cone relative to the axon shaft was demonstrated to be a result of differences in active transport speeds between anterograde- and retrograde-moving mRNAs, rather than a bias in the frequency of occurrence of the two opposing motions as suggested in a previous work studying the dynamics of *oskar* mRNAs in *Drosophila* oocytes by using the MS2 system (Zimyanin et al., 2008). The findings linking heterogeneous mRNA distributions and dynamics in the axons could provide mechanistic insight into the spatial regulation of axonal protein synthesis through mRNA trafficking.

## Combining MBs and the Clustered Regularly Interspaced Short Palindromic Repeats (CRISPR) System for Dynamic Imaging of Single Genomic Loci

**Figure 5:**
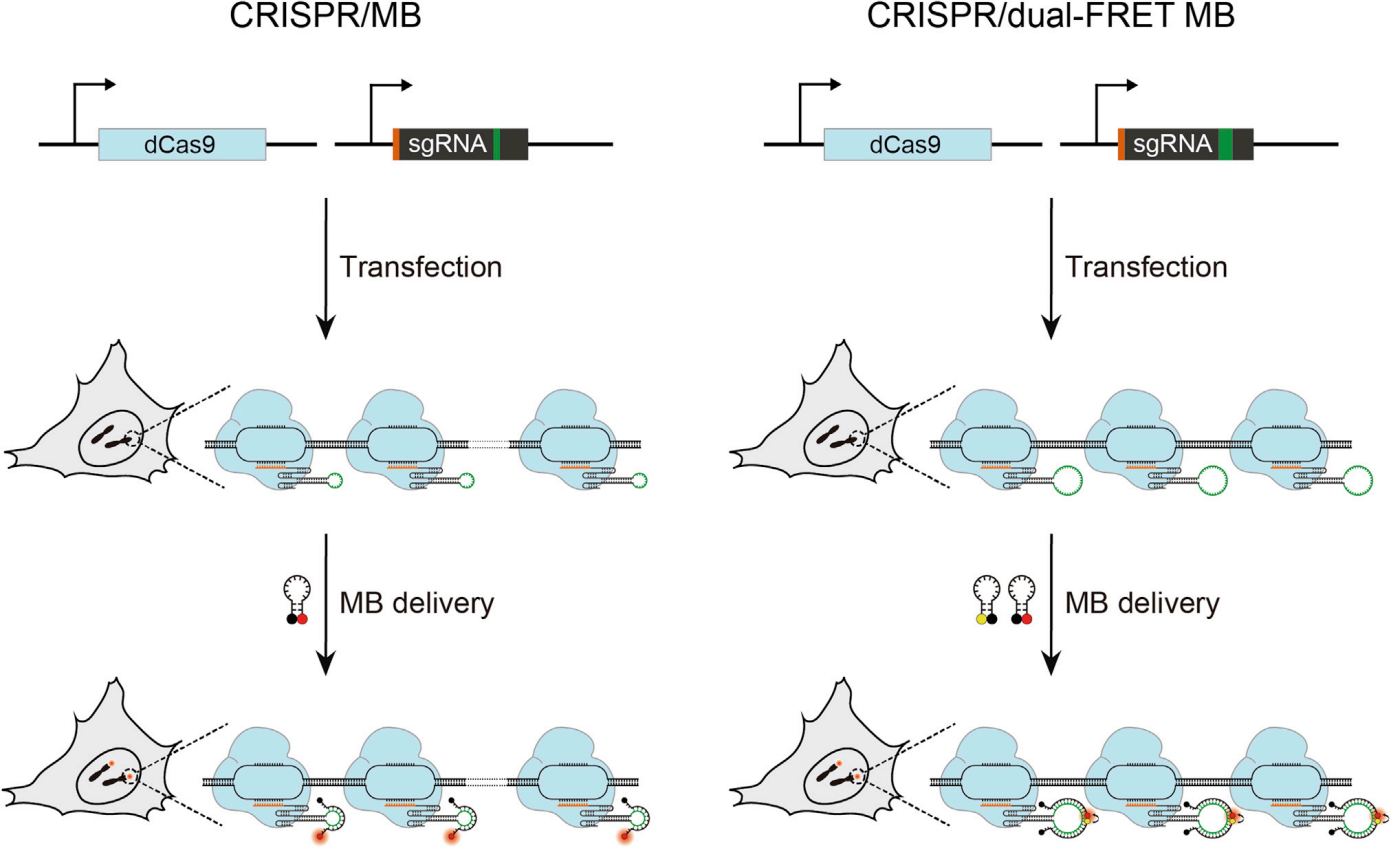
Imaging Single Genomic Loci Based on the Combined Use of MBs and the CRISPR System To image a specific genomic locus, dCas9 and sgRNA modified to harbor a unique sequence (green) that can be bound by either a single MB (for CRISPR/MB) (left panel) or dual-FRET MB (for CRISPR/dual-FRET MB) (right panel) are first co-expressed in cells (through plasmid transfection). After sufficient time is given to allow formation of dCas9-sgRNA complexes and their binding to the target sites specified by the sgRNA spacer sequence (orange), MBs are delivered into cells. Collective hybridization of MBs to dCas9-sgRNA complexes bound to adjacent sites within the target genomic locus can illuminate the locus as a single bright spot.

Besides imaging single RNA dynamics, the ability to visualize the dynamics of single genomic loci is also of critical importance, as many biological processes, including cell differentiation, development, and metastasis, are intimately orchestrated by variations in the spatiotemporal organization of genomes (Misteli, 2007; Bonev and Cavalli, 2016). However, unlike RNA, genomic DNA predominantly exists in the double-stranded form *in vivo*, making neither strand accessible to MBs or other oligonucleotide probes. Inspired by the fact that a specific genomic locus can be bound by the nuclease-deactivated version of the CRISPR-associated protein 9 (dCas9) in a single guide RNA (sgRNA)-mediated fashion (i.e., the CRISPR/dCas9 system) (Jinek et al., 2012; Qi et al., 2013; Wu et al., 2019), our group has recently developed an MB-based genomic imaging approach, termed CRISPR/MB, by modifying the sgRNA scaffold to contain a unique MB target sequence without impacting dCas9 binding and genomic targeting (Wu et al., 2018). Hybridization of MBs to dCas9-sgRNA complexes tiling across the target genomic sequences within a specific locus could illuminate the locus collectively as a single bright spot under epifluorescence microscopy (Figure 5). Additionally, the flexibility in MB target sequence and fluorophore/quencher pair selections made it possible to simultaneously illuminate multiple distinct genomic loci. More recently, we showed that the sensitivity of the CRISPR/MB system for genomic imaging could be further enhanced if the sgRNA is modified to accommodate two distinct MBs to allow dual-FRET MB-based imaging (i.e., CRISPR/dual-FRET MB) (Mao et al., 2019) (Figure 5). Only three unique sgRNAs were required to render a non-repetitive genomic locus visualizable by CRISPR/dual-FRET MB under epifluorescence microscopy, with the total molecular weight of a single CRISPR/dual-FRET MB imaging complex (~242 kDa) being only 14% of the total molecular weight of a single CRISPR imaging complex that is tethered to 32 FPs through the MS2 system (~1.76 MDa) (Qin et al., 2017) with comparable sensitivity. Clearly, CRISPR/dual-FRET MB has the potential to provide more accurate reflection of chromatin dynamics compared with an FP-based system and may be a valuable tool for elucidating chromatin dynamics across a range of different spatial and temporal scales.

## Overcoming Challenges in MB-Based Imaging

Despite the great progress made in the MB technology, several challenges still remain to be overcome before MBs can be truly reliable in furthering our understanding of the molecular basis underlying normal physiology and pathophysiology. Below, some of these challenges are discussed and their potential solutions are provided.

## Background Fluorescence

One crucial design advantage of MBs over conventional single-stranded unstructured (linear) probes is the self-quenchability in the absence of target hybridization, enabling sensitive live-cell imaging without the need to wash away unbound probes. However, due to general imperfect quenching of the fluorophores by currently available quenchers, MBs can still emit fluorescence in the closed configuration even if they are completely resistant to nonspecific opening. Therefore, when the use of MBs at a high concentration is required (i.e., within the nanomolar range), as is the case when intracellular RNA is imaged, unbound MBs may accumulate within a detection region and the resulting background signal may mask the signal arising from specific MB-target hybridization within the region. We should mention that the detectable single β-actin mRNA dynamics with only two MBs in the growing axon reported by Turner-Bridger et al. (2018) is a special case that is not easily attainable in the soma as well as commonly used nonpolarized model cell systems. This is because β-actin mRNAs can travel much more rapidly than unbound MBs along the axon so that within an axonal region of interest MB-labeled β-actin mRNAs could be detected against low background fluorescence emitted by a small amount of free unbound MBs. To reduce the impact of imperfect quenching in a more general model cell system, one strategy has been ratiometric imaging (Huang et al., 2018), which compares the fluorescence signal of the MB with that of an optically distinct reference probe to allow differentiation between bound and unbound MBs (Bratu et al., 2003; Chen et al., 2007, 2010). Alternatively, the dual-FRET MB approach can be used, as the approach is insensitive to signals emitted by single MBs (Bratu et al., 2003; Santangelo et al., 2004). Despite these advances, both strategies require using two distinct fluorescence channels, making them less suitable for multiplexed imaging. Perhaps a more direct approach is to enhance quenching by using multiple quenchers to pair with one fluorophore, i.e., a super-quencher MB design (Yang et al., 2005; Lovell et al., 2010; Ryazantsev et al., 2014). However, a super-quencher MB could potentially emit a low fluorescence signal even when it is opened upon target hybridization compared with the conventional single quencher MB design, particularly when the MB loop domain is designed to be short to enhance the binding specificity for the target RNA (Tsourkas et al., 2003a).

## Off-Target Binding

Based on studies in the test-tube environment, MBs have been reported to be capable of differentiating perfect targets from targets with a single base mismatch compared with single-stranded linear probes, as the stem domain can impose an energy barrier on loop-target hybridization (Tyagi et al., 1998; Bonnet et al., 1999). More recently, this attribute of MBs has been confirmed in cell-based studies where known concentrations of MBs and synthetic targets (perfect or mismatched) were microinjected into living cells and the resulting MB fluorescence intensities were compared (Chen et al., 2016; Turner-Bridger et al., 2018). However, whether the similar specificity holds true when using MBs to image RNAs transcribed in the native cellular environment has remained to be established. This is in part because the expression levels of a target RNA and its competitor RNAs (i.e., RNAs containing sequences similar to the MB target sequence) within a cell are largely unknown and can vary significantly with time, owing to the fact that gene expression occurs stochastically and transcription rates exhibit pulsatile variations (Chubb et al., 2006; Raj et al., 2006; Raj and van Oudenaarden, 2008; Larson et al., 2009; Muramoto et al., 2012; Rodriguez et al., 2019). Thus, in addition to its bona fide target, an MB could also hybridize to the competitors, particularly when the latter are expressed at high levels to shift the binding equilibrium toward the formation of mismatched duplexes, generating a false-positive signal independent of nuclease digestion or nonspecific protein binding. By all means, it is recommended that the target sequence chosen for MB imaging should be as unique as possible in the model cell systems used (e.g., determined through Basic Local Alignment Search Tool) and that the resulting MB signal should be validated by approaches that can be performed directly on the same target RNA labeled by MBs, such as single-molecule fluorescence *in situ* hybridization (Raj et al., 2008).

## Target Selection

Besides being unique, the target RNA region for an MB should be free of secondary structure. Moreover, although it is thought that MBs exhibit improved binding capacity for their target RNAs when compared with RNA-binding proteins and thus may displace the bound proteins from target sites to result in successful labeling (Oliveira et al., 2020), displacing proteins from their cognate binding sites may disrupt the normal activities of the target RNA (Glisovic et al., 2008). Therefore, we also recommend that the target RNA region for an MB should not be engaged in interactions with its cognate binding partners. To date, accurate prediction of the structure of an RNA as well as its variations with space and time within the native cellular environment has been a formidable challenge. For simplicity, selection of potential target RNA regions has conveniently been achieved based on structures predicted by computational algorithms (Zuker, 2003; Bayer et al., 2019). However, such algorithms could only provide somewhat reliable predictions and are particularly not accurate if the RNA of interest is greater than 400 nucleotides in length (Reeder et al., 2006). Alternatively, owing to the similar target selection requirement, RNA regions potentially targetable by MBs may be inferred from previously published siRNA target regions, but the selection is limited to the availability of published results and regions available for MB labeling and for siRNA targeting may not always coincide (Rhee et al., 2008). To improve target selection, transcriptome-wide structure probing-based approaches that can be applied to cell samples, such as selective 2′-hydroxyl acylation analyzed by primer extension (SHAPE) (Merino et al., 2005; Spitale et al., 2013), may unravel target regions not identifiable by computational algorithms. Nonetheless, at this juncture, these approaches can only provide a single predominant conformation of an RNA pertaining to a particular biological state.

## Outlook and Future Directions

With the development of efficient probe delivery strategies, methods to minimize nonspecific signals, as well as high-resolution fluorescence microscopy, we have witnessed the transition of MBs from being only useful for reporting RNA activity based on bulk fluorescence to a highly sensitive tool capable of illuminating the spatiotemporal dynamics of specific RNAs and even genomic loci at the single-molecule level. With further possible approaches for enhancing the detection sensitivity and accuracy, accompanied by the use of more sophisticated imaging techniques such as multispectral imaging (Cohen et al., 2018) and super-resolution microscopy (Sahl et al., 2017), we anticipate that MBs can be reliable in promoting new discoveries in various biological research fields. For example, in cancer biology, MBs could help elucidate the molecular mechanism by which oncogenic microRNAs are packaged into extracellular vesicles (EVs) within cancer cells and study how the resulting EVs facilitate the spread of cancer (Shurtleff et al., 2018). In virology, MBs could be used to measure the rate of viral RNA synthesis and to study the pathway leading to the formation of infectious virions (Freed, 2015; Bieniasz and Telesnitsky, 2018; Rein, 2019; Cascella et al., 2020). Last but not least, in epigenetics research, MBs could be combined with CRISPR-based or other genomic imaging platforms to investigate the dynamic interactions between lncRNAs and specific genomic loci, which are increasingly realized to be crucial in the regulation of genome architecture and gene expression (Engreitz et al., 2016; Yao et al., 2019).
